# Association of Hip Bone Mineral Density and Body Composition in a Rural Indian Population: The Andhra Pradesh Children and Parents Study (APCAPS)

**DOI:** 10.1371/journal.pone.0167114

**Published:** 2017-01-06

**Authors:** Mika Matsuzaki, Bharati Kulkarni, Hannah Kuper, Jonathan C. Wells, George B. Ploubidis, Poornima Prabhakaran, Vipin Gupta, Gagandeep Kaur Walia, Aastha Aggarwal, Dorairaj Prabhakaran, George Davey Smith, Kankipati Vijaya Radhakrishna, Yoav Ben-Shlomo, Sanjay Kinra

**Affiliations:** 1 Department of Non-communicable Disease Epidemiology, London School of Hygiene and Tropical Medicine, London, United Kingdom; 2 National Institute of Nutrition, Indian Council of Medical Research Tarnaka, Jamai-Osmania, Hyderabad, India; 3 Department of Clinical Research, London School of Hygiene and Tropical Medicine, London, United Kingdom; 4 Childhood Nutrition Research Centre, UCL Institute of Child Health, London, United Kingdom; 5 Department of Population Health and Statistics Centre for Longitudinal Studies, Institute of Education, University of London, London, United Kingdom; 6 Public Health Foundation of India, Gurgaon, Haryana, India; 7 Department of Anthropology, University of Delhi, Delhi, India; 8 Centre for Chronic Disease Control, Gurgaon, Haryana, India; 9 MRC Integrative Epidemiology Unit (IEU) at the University of Bristol, School of Social and Community Medicine, University of Bristol, Bristol, United Kingdom; National Center for Cell Science, INDIA

## Abstract

**Background:**

Fat mass is variably associated with bone mass, possibly due to differential mechanical and biological effects of fat mass. We examined the association of fat mass with bone mass in a lean population.

**Objective:**

To investigate association between hip bone mineral density and fat and lean mass in a cross-sectional study from southern India.

**Design:**

The Andhra Pradesh Children and Parents Study is a prospective cohort study in Hyderabad, India. In 2009–2012, the study collected data on anthropometric measures, bone mineral density (BMD), fat mass, and lean mass measured by dual-energy x-ray absorptiometry, and socioeconomic data of the adult participants (n = 1760; mean age = 34.9 years old for women; 2130 and 32.3 for men).

**Results:**

The median BMI (kg/m^2^) was 20.1 kg/m^2^. Women had relatively higher fat mass as compared to men. In models adjusted for lean mass, there was an association between hip bone mineral density and fat mass in women (β (95% confidence interval): premenopausal 0.025 (0.006 to 0.045); postmenopausal 0.045 (0.014 to 0.076)) but not in men (0.001 (-0.012 to 0.0014)). The association between hip BMD and fat mass was stronger in postmenopausal than premenopausal women. Hip BMD was consistently associated with lean mass, in both men and women.

**Conclusions:**

In this relatively lean population, lean mass was more consistently associated with hip BMD than fat mass. Weight gain through lean mass improvement may be a more reliable public health strategy for strengthening bone health in transitional settings.

## Introduction

Osteoporotic fractures are associated with morbidity and mortality [1]. A study estimated that the number of hip fractures will rise to 6.3 million globally by 2050 [2]. Hip bone mineral density (BMD) is a predictor of overall risk of fractures in later life [3]. Body mass is one of the key determinants of hip bone mineral density [4].

Fat mass is one of the main components of body mass. On one hand, sheer mechanical loading of fat mass stimulates bone formation [5,6]. However, *in vivo* and *in vitro* studies have suggested negative effects of fat on bone mass accrual through several biological mechanisms [5–8]. Leptin, an adipocyte hormone, was shown to have an anti-osteogenic property in mice [9]. Shared precursor stromal cells suggest competitive cell lineages between osteogenesis and adipogenesis [10]. Peroxisome proliferator-activated receptor (PPAR) γ pathway is a key regulator of adipogenesis and also an inhibitor for osteoblastogenesis [7].

Epidemiological studies have shown inconsistent results on association between bone and fat mass [11–14]. Many studies examined this association in the context of obesity and osteoporosis partially because of concerns for higher fracture rates among obese individuals [15]. However, it is possible that the balance between mechanical and biological mechanisms may vary depending on the combination of body size and composition. Asians have been shown to have higher proportion of fat mass at lower body mass index (BMI) [16]. The patterns of association between fat and bone mass in the Indian population may therefore be distinct from the European and American populations. There have been few large-scale studies examining this association in the Indian population.

The Andhra Pradesh Children and Parents Study (APCAPS) is a prospective cohort study from southern India. The study population has been undergoing a nutritional and epidemiological transition due to urbanization over the past decade; as a result, this population has a wide variation in body sizes and compositions. The current study assessed how fat and bone mass may be associated in this transitional rural population.

## Methods

### Ethics statement

The study received approvals from the ethics committees of the NIN (Hyderabad, India), the Indian Council of Medical Research (ICMR), Centre for Chronic Disease Control, and London School of Hygiene and Tropical Medicine (London, UK). Approval was also sought from the village heads and their panchayats in each of the 29 villages. Written informed consent or witnessed thumbprint if illiterate was obtained from the participants prior to their inclusion in the study.

### Study design

The analyses in this study used data from two waves of data collection (2009–2010; 2010–2012) of the APCAPS study, a prospective cohort study established through long-term follow up of the Hyderabad Nutrition Trial (HNT). The HNT studied impact of the Integrated Child Development Services scheme, a national community outreach programme providing food supplementation along with health, hygiene, and nutrition education, immunization, anemia control, and basic health care to pregnant and lactating women and children under the age of six years [17]. A detailed description of the initial trial (HNT) and the first wave of data collection (the first follow-up of the HNT, 2003–2005) has previously been published [17,18].

Since the second and third waves of data collection (W2/3) were conducted within a relatively short period of time (2009–2012), the analyses in this manuscript used combined data from these two waves of data collection. W2/3 examined markers for chronic diseases affecting cardiovascular, musculoskeletal, and mental health. All consenting participants underwent dual-energy x-ray absorptiometry (DXA) measurements at the National Institute of Nutrition (NIN), Hyderabad and physical measurements at NIN (W2) or the village clinics (W3). In cases where participants attended both waves of data collection, the data from the third wave were used, unless there were major artifacts in the DXA scans from W3, which prompted the use of data from W2. The current manuscript analyzed data on the adult participants only (18 years old and above).

Of the 7375 participants of the second/third wave of data collection whose age, sex, height, and weight information were available, 4251 participants (58%) underwent DXA scans during W2/3. Of those, scans without major artifacts were available in 4243 (99.8%) participants for hip BMD. 97.5% of the DXA participants also had scans without major artifacts for whole-body estimation of fat and lean mass. Information on the other descriptive variables were available for ≥99% of the DXA participants. In total, 1200 premenopausal women, 560 postmenopausal women, and 2130 men who had complete data for hip BMD, whole body composition and demographic data were include in the regression models.

### Measurements

#### Questionnaire data

A semi-structured questionnaire was administered to all participants by a trained interviewer. A subset of questions (14/29) from the Standard of Living Index (SLI) in the National Health Family Survey-2, a summary measure of household level asset-based scale devised for Indian surveys, was used to estimate socioeconomic position as joint family structures are common in rural India [19]. We collected information on the quality of house, toilet facilities, source of lighting and drinking water, ownership of clock, radio, television, bicycle, motorcycle, car, refrigerator, telephone, and agricultural land. These items were weighted to give a maximum score of 34, using weights developed by the International Institute of Population Science in India [19]. Occupation was classified into four categories: students, manual employment, professional employment, and unemployment. Current tobacco use was defined as smoking, chewing, or snuffing tobacco in the last 6 months. Menopausal status was set as a binary variable (yes or no).

#### Anthropometric data

Weight was measured to the nearest 0.1kg with a digital SECA balance and standing height was measured to the nearest 1 mm with a plastic stadiometer (Leicester height measure). Measurements were taken twice and the average of two values was used in the analysis. Body mass index (BMI) was calculated as weight (kg) / height (m^2^).

#### DXA scanning

Bone density and body composition measures were made by DXA using a Hologic Discovery A densitometer. The whole body scan was performed with the participant supine on the scanning bed with their arms resting by their sides. Women suspected of pregnancy were excluded from DXA scanning and the scans were taken only after confirming the negative pregnancy by conducting urine pregnancy test. Standard Hologic software options were used to define regions of the body (head, arms, trunk, and legs). The coefficients of variation were determined to be 0.7% for hip bone mineral density (BMD), 1.3% for LS BMD, and 0.9% for whole-body BMD (n = 30). Scans were coded for artifacts by a visual inspection and those hip scans with major movement and foreign objects as well as incomplete scans were excluded from the analyses of hip BMD (g/cm^2^). Whole body scans with major movements and incomplete scans were counted as artifacts and removed from analysis with fat and lean mass. Bone mineral content (BMC in g) was calculated from DXA-measured BMD (g/cm^2^) and bone area (cm^2^) for total hip. Fat to lean mass ratio (FLR: fat mass / lean mass^x^) was calculated using the allometric coefficients from sex-stratified models regressing log-transformed fat mass upon log-transformed lean mass as *x* (x = 1.57 for women; 1.66 for men). FLR was multiplied by 100 to improve clarity. Total fat and lean mass (kg) were estimated from whole-body scans. Osteopenia and osteoporosis were defined based on the reference BMD values measured on Hologic DXA machines in healthy Indian young adults (Hip BMD mean (sd): Women = 0.901 (0.111); Men = 0.988 (0.131)) [20].

### Statistical analysis

Descriptive statistics were calculated for premenopausal women, postmenopausal women, and men separately. Comparison between premenopausal and postmenopausal women and women and men were made using Student t-test for the continuous variables with normal distributions (height, weight, lean mass, wbPA, and SLI), Wilcoxon rank-sum test for the continuous variables with non-normal distributions (age, fat, FLR, and wbPA), and χ2 test for the categorical variables (BMI categories, tobacco use, vegetarianism, and occupation) with appropriate degrees of freedom. Tukey’s honest significant difference test was also performed to assess BMD differences between age groups within each sex group.

The associations between hip BMD and fat mass, lean mass, and FLR were examined in multilevel regression models that accounted for household-level clusters. Three-level nested multilevel models to adjust for both village and household-level clusters were considered but the small intraclass correlations for the village level suggested that adjustment for the household-level clustering alone was sufficient. All models were stratified by sex. There was evidence of an interaction between fat mass and menopausal status; therefore, regression models for women were stratified by menopausal status. Fat mass (kg) was log-transformed as its distribution was positively skewed. Hat, PRESS, and Cook statistics were examined to remove outliers for regression models.

Model 1 assessed association between hip BMD and fat mass, adjusting for lean mass, age, height, SLI. Model 2 (S1 Table) examined association between hip BMD and FLR, adjusting for age, height, and SLI. Model 3 (S1 Table) further adjusted Model 2 for weight. Further adjustment for other potential confounders (vegetarianism and current tobacco use) did not materially change the results and therefore parsimonious models are presented.

All analyses were conducted using R, version 3.1.1 and multilevel modeling was done with nlme version 3.1–118.

## Results

Table 1 summarizes the key characteristics of the participants. Although the average BMIs were similar between women and men, women had higher fat to lean mass ratio than men. Current tobacco use was more common among men. Premenpausal and postmenopausal women differed in all characteristics except for FLR. There were few vegetarians in this community. As shown in Table 2, young women (20–29 years old) in the APCAPS community had remarkably lower hip BMD in comparison Indian reference values for women in the same age group (0.901±0.111 g/cm^2^) [20]. Men had higher hip BMD than women across all ages.

**Table 1:** Characteristics of the participants of the Andhra Pradesh Parents and Children Study (2009–2012).

	Premenopausal women	Postmenopausal women	Men
	(n = 1200)	(n = 560)	(n = 2130)
	mean(sd)	mean(sd)	mean(sd)
age (year)	29.3(9.9)	46.8(6.9)	32.3(14.7)
height (cm)	152.4(5.5)	150.2(5.4)	165(6.5)
weight (kg)	47.6(9)	48.5(9.2)	55.3(9.9)
BMI (kg/m^2^)	20.5(3.6)	21.5(3.7)	20.3(3.2)
Underweight (n)	407	136	724
Normal (n)	650	335	1209
Overweight/obesity (n)	143	89	197
Fat mass (kg)	14.7(5.1)	15.9(5.3)	10.1(4.7)
Lean mass (kg)	31.6(4.5)	31.7(4.5)	43.6(6.2)
FLR	6.19(1.49)	6.66(1.56)	1.88(0.69)
wbPA (hours)	92.6(122.4)	156.9(143.4)	143.5(119)
vegetarian (n)			
yes	52	10	42
no	1148	550	2088
tobacco use (n)			
current	63	152	683
never/former	1137	408	1447
SLI	17.7(4.8)	16.6(4.7)	18.2(4.5)
occupation (n)			
student	170	2	444
employed: manual	638	474	1561
employed: professional	20	0	45
unemployed	372	84	80

BMI = body mass index; FLR = fat to lean mass ratio; SLI = Standard of Living Index; wbPA = weight-bearing physical activity.

All values are mean (standard deviation) except for occupation (%), vegetarian (n), menopause status (n), and tobacco use (%).

FLR for women: fat mass / lean mass^1.57^ x 100; for men: fat mass / lean mass ^1.66^ x 100

Current tobacco use included smoking, chewing, or snuffing tobacco in the last 6 months; former users stopped using tobacco products 6 months ago or more.

Comparison between premenopausal and postmenopausal women and women and men were made using t-test for the continuous variables with normal distributions (height, weight, lean mass, wbPA, and SLI), Wilcoxon rank-sum test for the continuous variables with non-normal distributions (age, fat, FLR, and wbPA) and χ2 test for the categorical variables (BMI categories, tobacco use, vegetarianism, and occupation) with appropriate degrees of freedom. p-values for comparison between premenopausal and postmenopausal women were <0.001 for all variables except for weight (p = 0.04) and FLR (p = 0.79); p-values for comparison between women and men were all <0.001.

**Table 2:** Description of mean hip bone mineral density, osteopenia (%), and osteoporosis (%) by sex and age groups for the participants of the Andhra Pradesh Parents and Children Study (2009–2012).

	Women				Men				
age	n	BMD (g/cm^2^)	osteopenia (%)	osteoporosis (%)	n	BMD (g/cm^2^)	osteopenia (%)	osteoporosis (%)	p^a^
20–29	666	0.84(0.1)	28.4	0.9	1164	0.95(0.11)	14.6	0.3	<0.001
30–39	204	0.86(0.1)	24.5	0.5	108	0.93(0.11)	25	0	<0.001
40–49	648	0.84(0.11)	30.9	1.1	253	0.92(0.12)	29.2	1.6	<0.001
50≤	248	0.75(0.12)	51.2	14.9	516	0.89(0.12)	39.7	3.3	<0.001

BMD = bone mineral density (g/cm^2^).

All participants who had at least age and hip BMD data and were 20 years old or older were included in this table.

Osteopenia is defined as 1 to 2.5 standard deviations (sd) and osteoporosis as more than 2.5 sd below peak bone mass in a healthy Indian population for each sex (reference hip BMD: women = 0.901±0.111; men = 0.988 ±0.131g/cm^2^).

^a^ Student t-test comparing hip BMD between sex within each age group was conducted.

Tukey’s honest significant difference test showed that hip BMD in the 20–30 and 30–40 year old groups were similar and higher than the ≥50 year old groups for both men and women (p≤0.001).

In the multilevel models adjusting for age, height, and SLI, fat mass (in women only) and lean mass were positively associated with hip BMD (Table 3). There was no clear evidence of association between hip BMD and fat mass in men adjusting for lean mass. There was clear and consistent evidence for a positive association between hip BMD and lean mass. In models examining association between hip BMD and fat to lean mass ratio (S1 Table), FLR was negatively associated with hip BMD upon adjustment for total weight. Adjusting models for other potential confounders such as serum calcium and phosphorus levels, vegetarianism, tobacco use, and parity (for women only) did not materially change the results on association between hip BMD and fat mass.

**Table 3:** Association of hip bone mineral density (BMD) and fat and lean mass in the participants of the Andhra Pradesh Parents and Children Study (2009–2012).

		Model 1	
		β	p
		95% CI	
**Pre-menopausal women**			
	Fat mass (kg)	0.025	0.01
		(0.006 to 0.045)	
	Lean mass (kg)	0.009	<0.001
		(0.008 to 0.011)	
**Post-menopausal women**			
	Fat mass (kg)	0.045	0.008
		(0.014 to 0.076)	
	Lean mass (kg)	0.01	<0.001
		(0.007 to 0.012)	
**Men**			
	Fat mass (kg)	0.001	0.92
		(-0.012 to 0.014)	
	Lean mass (kg)	0.01	<0.001
		(0.009 to 0.011)	

Sample size: n = 1200 (pre-menopausal women); n = 560 (post-menopausal women); n = 2130 (men).

CI = confidence interval.

All models are multilevel models adjusting for household level clustering. ε_ij_ and υ_j_ are errors terms for multilevel regression models accounting for individual and household level differences:

Model: HIP BMD = β_0_ + β_1_ FAT MASS + β_2_ LEANMASS + β_3_ AGE + β_4_ HEIGHT + ε _ij_ + υ_j_

Age (years); Height (cm); Fat and lean mass (kg)

Fat mass (kg) has been log-transformed.

## Discussion

Hip bone mineral density was low in this relatively lean population in rural India. There was a positive association between hip BMD and fat mass in women (who also had relatively higher fat to lean mass ratio), but not in men. Higher hip BMD was consistently associated with greater lean mass in this population.

### Comparison to previous studies

A number of epidemiological studies have examined the association between bone and fat mass: some showed no clear evidence of association while both positive and negative associations were also suggested [12,13,21–26]. As with many of the previous studies, we saw more consistent evidence for a positive effect of lean mass than fat mass [24,27]. Previous studies on Asian populations have shown attenuation of the association between BMD and fat mass upon adjustment for lean mass, similarly to our findings [24,25,27–29]. Our study also showed that for a given body size, lower fat to lean mass ratio may be associated with higher hip BMD, which suggests that both body mass and composition may be important determinants of healthy bone mass accrual. A large DXA study from China examined association between bone mass and body composition in a similarly lean population [12]. In unadjusted models, fat mass was positively associated with hip BMD, but on adjustment for body weight, fat mass became negatively associated with hip BMD, similar to the findings from our study.

Fat may have opposing effects on bone mass accrual through mechanical and biological mechanisms. Positive association between mechanical loading and bone mass accrual have been shown consistently in studies examining the effects of body mass and weight-bearing physical activity [4,6,30–33]. The mechanostat theory suggests that weight causes structural adaptation through local mechanical strain, which was supported by direct measurement of mechanical strain in live animals and humans [5,33]. On the other hand, *in vitro* and *in vivo* studies have suggested several biological mechanisms underlying the association between fat and bone mass. Osteoblasts and adipocytes originate in common stromal cells in bone marrow, suggesting plasticity between these two cell lineages [34]. Insufficiency in PPAR ***γ***, a key regulator for adipocyte differentiation, increased osteoblastogenesis *in vitro* and bone mass *in vivo* [7]. Leptin is a hormone produced by adipocytes, regulating both fat distribution and bone turnover through the hypothalamic neural networks [26].

The combined effect of mechanical and biological properties of fat on bone mass accrual is not well-established in epidemiological studies [35] and may differ depending on sex, menopausal status, and ethnicity. [36–38]. In our study, hip BMD was positively associated with fat mass in women, but not in men. Certain fat hormones suggested to be beneficial for bone mass accrual may be more strongly associated with bone mass accrual in women [23]. Another potential explanation may be that the amount of fat mass in men in this lean population was too low to detect its contribution to hip BMD in the presence of lean mass. In an Australian study, fat mass was also associated with hip BMD more consistently among women than men [39]. Interestingly, the positive association was only seen in lean men (BMI <25kg/m^2^); however, the male participants in this study, on average, had higher fat mass (24.9±9.3kg/m^2^) than our study population (10.3±9kg/m^2^) [39]. Such distinct combinations of body size and composition may have contributed to varying degrees of association between bone and fat mass in our study population and the Australian population. A study from the United States, where the men on average had greater fat mass and BMI than the men in this study population, showed no strong evidence for a positive association between BMD and fat mass and leptin in men while in women, adjustment for leptin attenuated the association between BMD and fat mass [23]. These studies suggest that sexual dimorphism may contribute to the varying degrees of association between fat and bone mass between women and men.

The association between hip BMD and fat mass was also slightly greater in postmenopausal than premenopausal women. Several studies have shown greater positive effects of fat mass in postmenopausal women although underlying biological mechanisms are not well-understood [38,40]. Adipose tissue is a key site for estrogen production in postmenopausal women [41]. Since estrogen has been suggested to play a role in the association between bone and fat mass, this may partially explain the difference in the strengths of the association between premenopausal and postmenopausal women [41,42].

It is important to note that this population had low hip BMD when compared to the values in a healthier Indian population [20]. One potential explanation for this finding is that although this rural community has become more developed and has been experiencing a nutritional transition over the past decade, modest gain in weight and lean mass during mid to late adulthood may not be able to fully mitigate adverse effects of undernutrition at younger ages when the majority of bone mass accrual occurs. Our previous study showed that gain in weight and lean mass in late adolescence and young adulthood was positively associated with bone mass (unpublished data) but it is currently unknown how long the window of opportunity to improve bone mass lasts.

### Strengths and limitations

This study is one of the few large-scale DXA studies examining the Indian population. The study subjects reside in an urbanizing rural community where there is a wide range of combinations in body sizes and compositions, allowing assessment of the association between hip BMD and fat mass in both underweight and overweight populations. The use of DXA provides more accurate estimation of fat and lean mass than anthropometric measurements.

There are some limitations in this study as well. The cross-sectional nature of the study does not allow causal inference. This community has been experiencing a nutritional transition due to urbanization over the past decade. There may be risk factors from the past that are contributing to current bone mass, although, in young adults, our previous studies did not find strong evidence for longitudinal effects of early life [43] and adolescent undernutrition [44] on bone mass during adulthood after controlling for current body mass. The study population was also lean compared to higher income countries [45]; therefore, our findings may not be generalizable to other populations but the findings may be of interest to other lean populations whose body compositions are more similar to this study population in India. Another limitation is the lack of data on fractures and fat hormones, which would be of clinical and biomedical importance.

### Conclusions

In this relatively lean population, hip BMD was associated with lean mass in both men and women, but hip BMD was associated with fat mass in women only. Weight gain through lean mass improvement may be a more reliable public health strategy for strengthening bone health in transitional settings.

## Supporting Information

S1 Table Multilevel regression models examining association between hip bone mineral density and fat to lean mass ratio in the participants of the Andhra Pradesh Parents and Children Study (2009–2012). (PDF)

## References

[1] Braithwaite RS, Col NF, Wong JB. Estimating Hip Fracture Morbidity, Mortality and Costs. J Am Geriatr Soc. 2003;51: 364–370. 12588580 10.1046/j.1532-5415.2003.51110.x

[2] Cooper C, Campion G, M DLJ Iii. Hip fractures in the elderly: A world-wide projection. Osteoporos Int. 1992;2: 285–289. 1421796 10.1007/BF01623184

[3] Johnell O, Kanis JA, Oden A, Johansson H, De Laet C, Delmas P, et al Predictive Value of BMD for Hip and Other Fractures. J Bone Miner Res. 2005;20: 1185–1194. 10.1359/JBMR.050304 15940371

[4] Felson DT, Zhang Y, Hannan MT, Anderson JJ. Effects of weight and body mass index on bone mineral density in men and women: the Framingham study. J Bone Miner Res Off J Am Soc Bone Miner Res. 1993;8: 567–573. 10.1002/jbmr.5650080507 8511983

[5] Frost HM. The Utah paradigm of skeletal physiology: an overview of its insights for bone, cartilage and collagenous tissue organs. J Bone Miner Metab. 2000;18: 305–316. 11052462 10.1007/s007740070001

[6] Hughes JM, Petit MA. Biological underpinnings of Frost’s mechanostat thresholds: the important role of osteocytes. J Musculoskelet Neuronal Interact. 2010;10: 128–135. 20516629

[7] Akune T, Ohba S, Kamekura S, Yamaguchi M, Chung U-I, Kubota N, et al PPARgamma insufficiency enhances osteogenesis through osteoblast formation from bone marrow progenitors. J Clin Invest. 2004;113: 846–855. 10.1172/JCI19900 15067317 PMC362117

[8] Pei L, Tontonoz P. Fat’s loss is bone’s gain. J Clin Invest. 2004;113: 805–806. 10.1172/JCI21311 15067310 PMC362126

[9] Takeda S, Elefteriou F, Levasseur R, Liu X, Zhao L, Parker KL, et al Leptin regulates bone formation via the sympathetic nervous system. Cell. 2002;111: 305–317. 12419242 10.1016/s0092-8674(02)01049-8

[10] Parhami F, Morrow AD, Balucan J, Leitinger N, Watson AD, Tintut Y, et al Lipid Oxidation Products Have Opposite Effects on Calcifying Vascular Cell and Bone Cell Differentiation : A Possible Explanation for the Paradox of Arterial Calcification in Osteoporotic Patients. Arterioscler Thromb Vasc Biol. 1997;17: 680–687. 9108780 10.1161/01.atv.17.4.680

[11] Liu J-M, Zhao H-Y, Ning G, Zhao Y-J, Zhang L-Z, Sun L-H, et al Relationship between body composition and bone mineral density in healthy young and premenopausal Chinese women. Osteoporos Int. 2004;15: 238–242. 10.1007/s00198-003-1536-7 14727013

[12] Hsu Y-H, Venners SA, Terwedow HA, Feng Y, Niu T, Li Z, et al Relation of body composition, fat mass, and serum lipids to osteoporotic fractures and bone mineral density in Chinese men and women. Am J Clin Nutr. 2006;83: 146–154. 16400063 10.1093/ajcn/83.1.146

[13] Reid IR. Relationships between fat and bone. Osteoporos Int. 2008;19: 595–606. 10.1007/s00198-007-0492-z 17965817

[14] Nguyen TV, Howard GM, Kelly PJ, Eisman JA. Bone mass, lean mass, and fat mass: same genes or same environments? Am J Epidemiol. 1998;147: 3–16. 9440393 10.1093/oxfordjournals.aje.a009362

[15] Compston JE, Watts NB, Chapurlat R, Cooper C, Boonen S, Greenspan S, et al Obesity Is Not Protective against Fracture in Postmenopausal Women: GLOW. Am J Med. 2011;124: 1043–1050. 10.1016/j.amjmed.2011.06.013 22017783 PMC4897773

[16] Wang J, Thornton JC, Russell M, Burastero S, Heymsfield S, Pierson RN. Asians have lower body mass index (BMI) but higher percent body fat than do whites: comparisons of anthropometric measurements. Am J Clin Nutr. 1994;60: 23–28. 8017333 10.1093/ajcn/60.1.23

[17] Kinra S, Krishna KR, Kuper H, Sarma KR, Prabhakaran P, Gupta V, et al Cohort Profile: Andhra Pradesh Children and Parents Study (APCAPS). Int J Epidemiol. 2013; dyt128. 10.1093/ije/dyt128 PMC4190511 24019421

[18] Kinra S, Rameshwar Sarma KV, Ghafoorunissa, Mendu VVR, Ravikumar R, Mohan V, et al Effect of integration of supplemental nutrition with public health programmes in pregnancy and early childhood on cardiovascular risk in rural Indian adolescents: long term follow-up of Hyderabad nutrition trial. BMJ. 2008;337: a605 10.1136/bmj.a605 18658189 PMC2500199

[19] The International Institute for Population Sciences. India National Family Health Survey 1998–99 (NFHS-2) [Internet]. [cited 11 Sep 2015]. http://dhsprogram.com/pubs/pdf/FRIND2/FRIND2.pdf

[20] Mukherjee A, Mathur A. Population based reference standards of peak bone mineral density of indian males and females. ICMR Bull. 2011; http://www.thefreelibrary.com/Population+based+reference+standards+of+peak+bone+mineral+density+of…-a0274521389

[21] Reid IR, Ames RW, Evans MC, Sharpe SJ, Gamble GD. Determinants of the rate of bone loss in normal postmenopausal women. J Clin Endocrinol Metab. 1994;79: 950–954. 10.1210/jcem.79.4.7962303 7962303

[22] Blum M, Harris SS, Must A, Naumova EN, Phillips SM, Rand WM, et al Leptin, Body Composition and Bone Mineral Density in Premenopausal Women. Calcif Tissue Int. 2003;73: 27–32. 14506951 10.1007/s00223-002-1019-4

[23] Thomas T, Burguera B, Melton LJ III, Atkinson EJ, O’Fallon WM, Riggs BL, et al Role of serum leptin, insulin, and estrogen levels as potential mediators of the relationship between fat mass and bone mineral density in men versus women. Bone. 2001;29: 114–120. 11502471 10.1016/s8756-3282(01)00487-2

[24] Douchi T, Kuwahata R, Matsuo T, Uto H, Oki T, Nagata Y. Relative contribution of lean and fat mass component to bone mineral density in males. J Bone Miner Metab. 2003;21: 17–21. 10.1007/s007740300003 12491089

[25] Ho-Pham LT, Nguyen ND, Lai TQ, Nguyen TV. Contributions of lean mass and fat mass to bone mineral density: a study in postmenopausal women. BMC Musculoskelet Disord. 2010;11: 59 10.1186/1471-2474-11-59 20346165 PMC2867833

[26] Rosen CJ, Klibanski A. Bone, Fat, and Body Composition: Evolving Concepts in the Pathogenesis of Osteoporosis. Am J Med. 2009;122: 409–414. 10.1016/j.amjmed.2008.11.027 19375545

[27] Wang MC, Bachrach LK, Van Loan M, Hudes M, Flegal KM, Crawford PB. The relative contributions of lean tissue mass and fat mass to bone density in young women. Bone. 2005;37: 474–481. 10.1016/j.bone.2005.04.038 16040285

[28] Namwongprom S, Rojanasthien S, Mangklabruks A, Soontrapa S, Wongboontan C, Ongphiphadhanakul B. Effect of fat mass and lean mass on bone mineral density in postmenopausal and perimenopausal Thai women. Int J Womens Health. 2013;5: 87–92. 10.2147/IJWH.S41884 23467695 PMC3589079

[29] Ho-Pham LT, Nguyen UDT, Nguyen TV. Association Between Lean Mass, Fat Mass, and Bone Mineral Density: A Meta-analysis. J Clin Endocrinol Metab. 2013;99: 30–38. 10.1210/jc.2013-3190 24384013

[30] Hannan MT, Felson DT, Dawson-Hughes B, Tucker KL, Cupples LA, Wilson PWF, et al Risk Factors for Longitudinal Bone Loss in Elderly Men and Women: The Framingham Osteoporosis Study. J Bone Miner Res. 2000;15: 710–720. 10.1359/jbmr.2000.15.4.710 10780863

[31] Taaffe DR, Snow-Harter C, Connolly DA, Robinson TL, Brown MD, Marcus R. Differential effects of swimming versus weight-bearing activity on bone mineral status of eumenorrheic athletes. J Bone Miner Res Off J Am Soc Bone Miner Res. 1995;10: 586–593. 10.1002/jbmr.5650100411 7610929

[32] Bailey DA, McKay HA, Mirwald RL, Crocker PR, Faulkner RA. A six-year longitudinal study of the relationship of physical activity to bone mineral accrual in growing children: the university of Saskatchewan bone mineral accrual study. J Bone Miner Res. 1999;14: 1672–1679. 10.1359/jbmr.1999.14.10.1672 10491214

[33] Meakin LB, Price JS, Lanyon LE. The Contribution of Experimental in vivo Models to Understanding the Mechanisms of Adaptation to Mechanical Loading in Bone. Front Endocrinol. 2014;5. 10.3389/fendo.2014.00154 PMC4181237 25324829

[34] Nuttall ME, Patton AJ, Olivera DL, Nadeau DP, Gowen M. Human trabecular bone cells are able to express both osteoblastic and adipocytic phenotype: implications for osteopenic disorders. J Bone Miner Res Off J Am Soc Bone Miner Res. 1998;13: 371–382. 10.1359/jbmr.1998.13.3.371 9525337

[35] Lanyon LE, Sugiyama T, Price JS. Regulation of bone mass: Local control or systemic influence or both? IBMS BoneKEy. 2009;6: 218–226.

[36] Lu H, Fu X, Ma X, Wu Z, He W, Wang Z, et al Relationships of percent body fat and percent trunk fat with bone mineral density among Chinese, black, and white subjects. Osteoporos Int. 2011;22: 3029–3035. 10.1007/s00198-010-1522-9 21243336

[37] Reid IR, Plank LD, Evans MC. Fat mass is an important determinant of whole body bone density in premenopausal women but not in men. J Clin Endocrinol Metab. 1992;75: 779–782. 10.1210/jcem.75.3.1517366 1517366

[38] Douchi T, Oki T, Nakamura S, Ijuin H, Yamamoto S, Nagata Y. The effect of body composition on bone density in pre- and postmenopausal women. Maturitas. 1997;27: 55–60. 9158078 10.1016/s0378-5122(97)01112-2

[39] Zhu K, Hunter M, James A, Lim EM, Walsh JP. Associations between body mass index, lean and fat body mass and bone mineral density in middle-aged Australians: The Busselton Healthy Ageing Study. Bone. 2015;74: 146–152. 10.1016/j.bone.2015.01.015 25652209

[40] Aloia JF, Vaswani A, Ma R, Flaster E. To what extent is bone mass determined by fat-free or fat mass? Am J Clin Nutr. 1995;61: 1110–1114. 7733036 10.1093/ajcn/61.4.1110

[41] Nelson LR, Bulun SE. Estrogen production and action. J Am Acad Dermatol. 2001;45: S116–124. 11511861 10.1067/mjd.2001.117432

[42] Riggs BL, Khosla S, Melton LJ. A unitary model for involutional osteoporosis: estrogen deficiency causes both type I and type II osteoporosis in postmenopausal women and contributes to bone loss in aging men. J Bone Miner Res Off J Am Soc Bone Miner Res. 1998;13: 763–773. 10.1359/jbmr.1998.13.5.763 9610739

[43] Matsuzaki M, Kuper H, Kulkarni B, Radhakrishna K, Viljakainen H, Taylor AE, et al Life-course determinants of bone mass in young adults from a transitional rural community in India: the Andhra Pradesh Children and Parents Study (APCAPS). Am J Clin Nutr. 2014;99: 1450–1459. 10.3945/ajcn.113.068791 24695898 PMC4021785

[44] Matsuzaki M, Kuper H, Kulkarni B, Ploubidis GB, Wells JC, Radhakrishna KV, et al Adolescent undernutrition and early adulthood bone mass in an urbanizing rural community in India. Arch Osteoporos. 2015;10: 232 10.1007/s11657-015-0232-5 26323265 PMC4554734

[45] Flegal KM, Shepherd JA, Looker AC, Graubard BI, Borrud LG, Ogden CL, et al Comparisons of percentage body fat, body mass index, waist circumference, and waist-stature ratio in adults. Am J Clin Nutr. 2009;89: 500–508. 19116329 10.3945/ajcn.2008.26847 PMC2647766

