# Supplementary material for: Association of Hip Bone Mineral Density and Body Composition in a Rural Indian Population: The Andhra Pradesh Children and Parents Study (APCAPS)
Source: PLoS One. 2017 Jan 6;12(1):e0167114. doi: 10.1371/journal.pone.0167114 (PMC5217858; doi:10.1371/journal.pone.0167114)
Supplement: S1 Table — (PDF) [file pone.0167114.s001.pdf]

Supplemental Material Table S1: Multilevel regression models examining association between hip bone mineral density and fat to lean mass ratio in the participatns of the Andhra Pradesh Parents and Children Study (2009-2012).

		Model 2<br>β<br>95% CI	p	Model 3<br>β<br>95% CI	p
Women (pre)	n = 1200				
	FLR	0.008<br>(0.004 to 0.011)	<0.001	-0.01<br>(-0.014 to -0.006)	<0.001
Women (post)	n = 560				
	FLR	0.014<br>(0.009 to 0.019)	<0.001	-0.009<br>(-0.014 to -0.003)	0.005
Men	n = 2248				
	FLR	0.01<br>(0.004 to 0.017)	0.003	-0.046<br>(-0.054 to -0.039)	<0.001

CI = confidence interval; FLR = fat to lean mass ratio

All models are multilevel models adjusting for household level clustering. ϵ_{ij} and υ_j are errors terms for multilevel regression models accounting for individual and household level differences.

Model 3: HIP BMD $= \beta_0 + \beta_1$FLR $+ \beta_2$AGE $+ \beta_3$HEIGHT $+ \epsilon_{ij} + \upsilon_j$

Model 4: HIP BMD $= \beta_0 + \beta_1$FLR $+ \beta_2$AGE $+ \beta_3$HEIGHT $+ \beta_4$WEIGHT $+ \epsilon_{ij} + \upsilon_j$

Age (years); Height (cm); Fat and lean mass (kg)

FLR for women: $\frac{\text{fat mass}}{\text{lean mass}^{1.57}} \times 100$; for men: $\frac{\text{fat mass}}{\text{lean mass}^{1.66}} \times 100$
